# Molecular biology: Self-sustaining chemistry

**DOI:** 10.1186/1752-153X-1-25

**Published:** 2007-10-30

**Authors:** Paul Wrede

**Affiliations:** 1 Charité-Universitätsmedizin Berlin, Institut für Molekularbiologie und Bioinformatik, Arnimallee 22, D-14195 Berlin, Germany

## Abstract

Molecular biology is an established interdisciplinary field within biology that deals fundamentally with the function of any nucleic acid in the cellular context. The molecular biology section in *Chemistry Central Journal *focusses on the genetically determined chemistry and biochemistry occuring in the cell.

How can thousands of chemical reactions interact smoothly to maintain the life of cells, even in a variable environment? How is this self-sustaining system achieved? These are questions that should be answered in the light of molecular biology and evolution, but with the application of biophysical, physico-chemical, analytical and preparative technologies.

As the Section Editor for the molecular biology section in *Chemistry Central Journal*, I hope to receive manuscripts that present new approaches aimed at better answering and shedding light upon these fascinating questions related to the chemistry of livings cells.

## Molecular biology in *Chemistry Central Journal*

At the outset, let me pose two important questions: Why is it necessary to have a molecular biology section within *Chemistry Central Journal*? Can molecular biology articles in this journal compete with contributions found in other excellent specialised molecular biology journals?

To answer these questions, let me start by first describing how the term molecular biology is understood today, before looking at the field in a chemical context.

## What is molecular biology?

Broadly, molecular biology aims to investigate the molecular genetics of all organisms, including man, by identifying mutants or genetic aberrations that are often the cause of diseases. Intense genetic studies on model organisms have cast light on the processes of morphological development, cell differentiation, and the control mechanisms governing organ (re)generation. Molecular biologists have generated their own specific methods to solve problems inherent in their field. Here of course I refer to techniques such as DNA recombination and nucleic acid amplification by PCR, whose functional basis is the manipulation of biological processes to gain deeper insight into complex molecular interactions either *in situ *or *in vitro*. The foundation of such techniques lies in the complementary nature of the genetic information contained in nucleic acids.

## Molecular biology and chemistry

The specific goals of molecular biology research are the discovery and more detailed understanding of the structure and function of genetic material, namely DNA and RNA, and in particular, how these molecules determine the smooth running of metabolic processes. Such investigations are more or less independent of the organism studied, as the conserved function of DNA and RNA in all organisms guarantees the continuation of this self-sustaining chemical system from one generation to the next.

In surveying the field of molecular biology one can only marvel at the fundamental similarities of many (bio)chemical reactions and processes – take, for example, the elaborate system of genes involved during the phase of early development. It is these similarities between complex processes that give us the possibility of categorizing molecular biology into several subdivisions. For example universal cellular processes like protein insertion and secretion or G-protein coupled signal transduction are self-dependent subdivisions of molecular biology.

## The cell – a chemical factory

It is always fascinating to consider that in a cell, thousands of different biochemical reactions can proceed under constant temperatures, such as in homoiothermic mammals and birds. In contrast, the complex metabolic network of poikilothermic organisms, like fish or plants, also include the same metabolic chemical reactions!

In cells several anabolic and catabolic reaction chains are separated from each other by 'compartments' like mitochondria, the endoplasmic reticulum, lysosomes, and many more. It is noteworthy that in all these separate locations, there occur different biochemical reactions, which are not however excluded from the intracellular mass transfer. For each 'compartment', transport proteins exist in the membranes responsible for the uptake and discharge of small molecules. Such transport can occur actively against the concentration gradient under ATP consumption or passively through protein channels in the membrane. Other organelles, such as the Golgi apparatus, exhibit a vesicular transport system for both generating new organelles like endosomes, and transporting proteins and small molecules within these vesicles. The directed transport processes between each 'compartment' occur in a finely tuned and controlled fashion. The genetic basis for these regulatory and control processes is one major source of interest in molecular biology research.

Thus to return to our initial question, as I have outlined, it is clear that molecular biology, with its special emphasis on the self-sustaining capacity of the cell's complex chemical reaction network, has a close relationship with the field of chemistry. All (bio)chemical reactions, including their regulation, have been transferred from one generation to the next for millions of years. Selectivity has resulted in the existence of highly conserved molecular processes; whilst conversely and interestingly, some metabolic pathways cannot be traced back to any specific origin owing to their occuring in only a few recent organisms.

## Major developments in molecular biology

The formative biological research activities of the last decade have been the genome sequencing projects of uni-cellular and multi-cellular organisms, including man. Surprisingly, the sequenced genomes have only shed limited light on increasing the understanding of the relationship between a gene and its expression and regulation. For instance, even the rudimentary question, 'what is a gene?', cannot yet be answered unambiguously.

Genome sequences between several human individuals can vary drastically, so it is often impossible to predict from the DNA-sequence alone who will be susceptible to a disease and who will remain healthy. All cellular organisms have in common a high variability, which includes, for example, gene order, protein sequence, glycosylation patterns and membrane constitution. This variability is further complicated by the fact that many different organisms share conserved metabolic concepts, such as the low energy delivery glycolysis pathway, the Krebs cycle for high chemical energy production, the genetic code or the protein translation apparatus. I think it is this dialectic principle, resulting from the dichotomy of great variability coexisting with strict conservation – not only on the molecular level but also on the organism level – that makes molecular biology a particularly fascinating discipline. Almost every year, progress in the field of molecular biology provokes at least a significant modification of existing concepts, and sometimes even a paradigm change.

## Techniques employed by molecular biologists

I mentioned at the outset techniques native to molecular biology, such as recombinant DNA and several other techniques like nucleic acid amplification by PCR, high resolution microscopy and very sensitive protein detection systems. These have helped accelerate the growth of our vast, but scattered, knowledge. Furthermore, significant progress in microarray techniques has delivered data on complex expression profiles, including post-translational modifications like glycosylation and phosphorylation. Here the differences in the patterns observed for normal and cancerous cells allow one to perform a classification of cellular subtypes. The expression profiles of cells from different tissues may even identify the activity status of metabolic pathways and their phylogenetic conservation. A combination of expression profiling data with confocal fluorescent microscopy and imaging processing allows one to locate protein expression activities within the cell. By combining all these data, one will be able to construct an image of the cell in time and space that will deepen our understanding of molecular interactions.

Metabolic processes can also be probed through the fine tuning of gene activities by silencing genes with small interfering RNA (siRNA) and microinterfering RNAi. Whilst the determination of the sequences and 3D structures of biological macromolecules have been significantly enhanced by many sophisticated biophysical techniques, also employed by chemists, such as MALDI and multidimensional NMR. Such techniques permit the identification and characterisation of many protein variants with unique catalytic centres and glycosylation/phosphorylation patterns that are specific for many different chemical reactions. Analytical techniques can now be performed almost quantitatively.

## Open access and molecular biology

Thousands of articles on molecular biology are published each year, meaning that inevitably experts can hardly keep a detailed track of research progress, even in their particular field. I am convinced that the principal benefits of open access publishing – that is to say, free and unhindered access to scientific research, rapid publication, authors' retention of copyright for their own work, free distribution and deposition of articles – will benefit the molecular biology community, and help cope with the huge flood of research in this field.

*Chemistry Central Journal's *parent company, BioMed Central, already operates a number of successful journals in the field of molecular biology – notably *Algorithms for Molecular Biology*, *BMC Molecular Biology*, and the *Journal of Molecular Signaling *– and related disciplines. In addition, *BMC Bioinformatics *complements molecular biology by concentrating on data mining and computer-based simulation systems, concepts which can help significantly support molecular biologists' experimental work.

## Conclusion

Of course a number of BioMed Central's other already-established open access titles, as well as the chemical biology section in *Chemistry Central Journal*, touch upon areas of interest to molecular biologists. Such overlap cannot be avoided, but rather serves to underline the interdisciplinary character of molecular biology.

However, that said, each of these journals or sections have their own particular focal points. For example, the chemical biology section in this journal focusses on small organic molecules and their interactions with target proteins; while the aim of the molecular biology section, however, will be to publish research highlights whose focus is the innate chemistry of the cell and its evolution, as well as the fascinating theme of gene regulation and expression in all organisms. Thus, within *Chemistry Central Journal *it is inevitable that both themolecular biology and chemical biology sections will complement and enrich each other. Of course, when submitting a manuscript, an author can select up to 3 sections by which to classify the article.

Finally, I also want to encourage molecular biologists to publish in *Chemistry Central Journal *because of the broad nature of our journal's audience – reaching from specialists in the field to those working in other fields but looking for cooperation partners to accelerate a common project – helped by the unhindered access to the journal's published research. All research presenting good science will be considered for publication and I am looking forward to receiving fresh and novel submissions.

I would like to close my commentary with two summatory statements: the first by Edmund B.Wilson: "*the key to every biological problem must finally be sought in the cell*" [1]; and the second by Theodosius Dobshanzky: "*nothing in biology makes sense except in the light of evolution*" [2].
